# Structure of the virulence-associated *Neisseria meningitidis* filamentous bacteriophage MDAΦ

**DOI:** 10.1073/pnas.2420157122

**Published:** 2025-06-20

**Authors:** Jan Böhning, Miles Graham, Mathieu Coureuil, Abul K. Tarafder, Julie Meyer, Xavier Nassif, Emmanuelle Bille, Tanmay A. M. Bharat

**Affiliations:** ^a^Structural Studies Division, Medical Research Council Laboratory of Molecular Biology, Cambridge CB2 0QH, United Kingdom; ^b^INSERM U1151, CNRS UMR8253, Institut Necker-Enfants Malades, Université Paris Cité, Paris F-75015, France

**Keywords:** cryo-EM, cryo-ET, inoviral phage, Neisseria, biofilm

## Abstract

*Neisseria meningitidis* is a human commensal bacterium that can opportunistically invade the bloodstream and cross the blood–brain barrier, where it can cause septicemia and meningitis. These diseases, if left untreated, can be lethal within hours. Hyperinvasive *N. meningitidis* strains often express a genomically encoded filamentous bacteriophage called MDAΦ, which promotes colonization of mucosal host surfaces to facilitate bacterial invasion. How this phage is organized and how it promotes biofilm formation and infection at the molecular level is unclear. Here, we present an electron cryomicroscopy structure of the MDA phage, showing that MDAΦ is a class I filamentous inovirus, with the major capsid protein (MCP) arranged within the phage as a highly curved and densely packed α-helix. Comparison with other filamentous bacteriophages offers clues about inoviral genome encapsidation mechanisms, providing a framework for understanding the evolutionary diversity of inoviruses. A disordered, N-terminal segment in the MCP presents hydrophobic patches on the surface of assembled phage particles, which, together with electron cryotomography data of phage bundles, furnishes a structural rationale for phage–phage interactions that were seen previously in an epithelium adhesion infection model of *N. meningitidis*. Taken together, our results shed light on the structure, organization, and higher-order assembly of a biomedically relevant phage encoded in the genome of a human pathogen. Molecular insights gleaned from this study increase our understanding of phage evolution, phage-mediated bacterial adhesion, and pathogenicity.

## Significance

*Neisseria meningitidis* is a human commensal bacterium found in the nasopharynx that can invade the bloodstream and cause meningitis, a potentially lethal disease. The most invasive *N. meningitidis* bacteria express a filamentous bacteriophage (inovirus) called MDAΦ that is encoded on the bacterial genome. MDAΦ particles are secreted by bacterial cells, forming adhesive phage bundles that promote formation of persistent *N. meningitidis* biofilms in the respiratory tract, which is a prerequisite of invasive disease. Here, we report the structure of the MDA phage using cryo-EM, providing molecular insights into inoviral capsid arrangement that is important for phage genome assembly, as well as phage bundling that is crucial for biofilm formation and infection.

*Neisseria meningitidis* is one of the major causes of bacterial meningitis, which can result in neurological damage and death within hours if untreated (1). *N. meningitidis* is found commensally in the throat, but can invade the bloodstream to cause septicemia and/or invasive meningeal disease after crossing the blood–brain barrier (2). To become septicemic, bacteria must first colonize the mucosal surfaces of the nasopharynx, after which they can invade the bloodstream (3–5). Hence, the factors promoting colonization of host mucosal surfaces are of particular importance for understanding disease progression (4–7).

Previous studies found that an eight kilobase genetic island encoding a filamentous bacteriophage, termed MDAΦ (short for meningococcal disease-associated bacteriophage), was a common feature in hyperinvasive populations of *N. meningitidis* (8, 9). Filamentous inoviruses such as MDAΦ are a genus of bacteriophages consisting of a single-stranded DNA (ssDNA) genome that is surrounded by an α-helical capsid, resulting in rod-like phage particles up to several micrometers in length but less than ten nanometers in diameter (10). While employing their host’s resources to replicate, filamentous phages are often continuously secreted into the environment without host lysis. In some cases, the phage is symbiotic with its bacterial host and secretion of the phage provides benefits to its host bacterium (11–13). For example, the human pathogen *Pseudomonas aeruginosa* encodes a phage, Pf4, which is secreted into the biofilm matrix to form protective assemblies around cells (14, 15). In the same vein, in *Vibrio cholerae,* the bacteriophage CTXΦ is a vital feature of virulent strains, carrying the cholera toxin genes responsible for key aspects of the etiology of cholera (16).

In *N. meningitidis,* MDAΦ particles, after being secreted into the extracellular milieu, assemble into filament bundles that promote cohesion between cells, thus promoting the formation of biofilms (17). In a previously proposed model, a layer of heavily piliated bacteria initiate adhesion to host epithelial cells, while subsequent layers of bacteria are encased in MDAΦ filaments that promote the formation of a thick biofilm (17). Promoting colonization of the mucosal surfaces in the nasopharynx increases the likelihood of subsequent invasion, suggesting a direct link between pathogenicity and the presence of the MDA bacteriophage (8, 9).

Despite the ubiquity of filamentous bacteriophages (18), and considering the important roles they play in many diseases (8, 12, 16), it is surprising that there is limited atomic structural information on inoviral phages themselves. Based on the symmetrical arrangement of the major capsid protein (MCP), inoviruses have been classified into two types: In class I inoviruses, the MCP is arranged helically with an additional pentameric rotational (C5) symmetry around the filament axis within the phage, while class II inoviruses have a helically arranged MCP with no additional rotational symmetry around the filament axis (10, 19, 20). All high-resolution structural work on inoviral capsids has been performed on a limited set of phages: the class II Pf phages (14), and the class I Ff phages (15, 21, 22), as well as on the Ff-related IKe phage (23). Little is known about the structure of filamentous bacteriophages outside of these families. It remains unknown which class of phage MDAΦ falls under, or what structural features of its capsid make it able to bundle into higher-order assemblies that promote biofilm formation during infection.

Here, we present the electron cryomicroscopy (cryo-EM) structure of native MDA inoviral bacteriophages isolated from *N. meningitidis*, revealing significant differences to previously solved inovirus capsid structures. Based on our structural data, together with previously available experimental sequencing data, we illuminate the links between capsid structure and genome arrangement, thus allowing us to classify inoviral genomes as circular or linear simply based on MCP sequence. We combine our analysis of the phage structure with electron cryotomography (cryo-ET) of phage bundles, which enables us to understand the rationale of phage–phage interactions, providing a structural basis for comprehending MDA phage-mediated virulence that depends on phage bundling.

## Results

### Cryo-EM Structure of the MDAΦ Capsid.

**Fig. 1.**
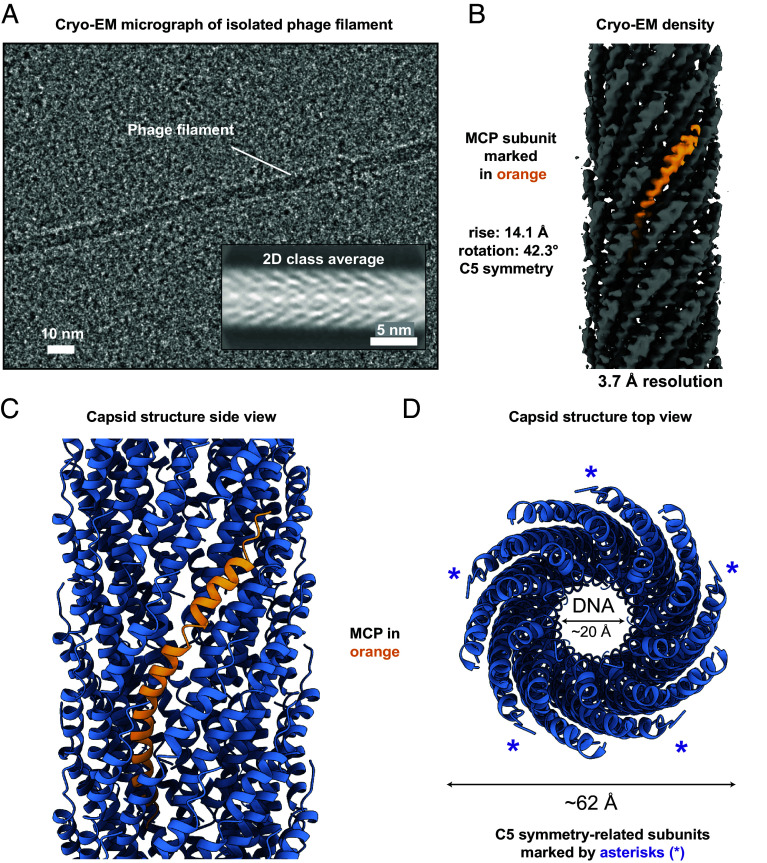
Cryo-EM structure of the filamentous inovirus MDAΦ. (*A*) Cryo-EM image of MDAΦ particles. *Inset*: 2D class average of MDAΦ, showing the arrangement of the α-helical capsid protein. (*B*) Cryo-EM density map, with a single MCP subunit marked. (*C* and *D*) Side and top view of the atomic model of the MDAΦ capsid, with a single MCP subunit marked in orange in panel *C*.

To determine the structure of the MDAΦ capsid, we natively expressed and isolated phages from *N. meningitidis* (Methods). MDAΦ particles were then concentrated for cryo-EM sample preparation. Images of the concentrated specimen show elongated rod-like particles consistent with filamentous bacteriophages from previous studies (14, 15, 23) (Fig. 1*A*). In addition to the phages, some vesicular contaminants were also observed on the cryo-EM grid that could not be removed by cesium chloride gradient centrifugation or ion exchange chromatography. Two-dimensional class averages of the phage particles show a helical arrangement of the phage capsid (Fig. 1 *A*, *Inset*). We estimated the helical parameters from two-dimensional class averages and employed real-space helical refinement (24) to solve the structure of the MDAΦ MCP to 3.7 Å resolution (Fig. 1 and *SI Appendix*, Figs. S1 and S2). The resulting cryo-EM map reveals α-helical MCPs packed into a tubular arrangement, with an overall diameter of ~62 Å and a central pore with a ~20 Å diameter (Fig. 1 and *SI Appendix*, Fig. S3). MCPs are arranged with a pentameric (C5) rotational symmetry around the filament axis, with a rise of 14.1 Å and 42.3° rotation per subunit, establishing MDAΦ as a class I inovirus.

**Fig. 2.**
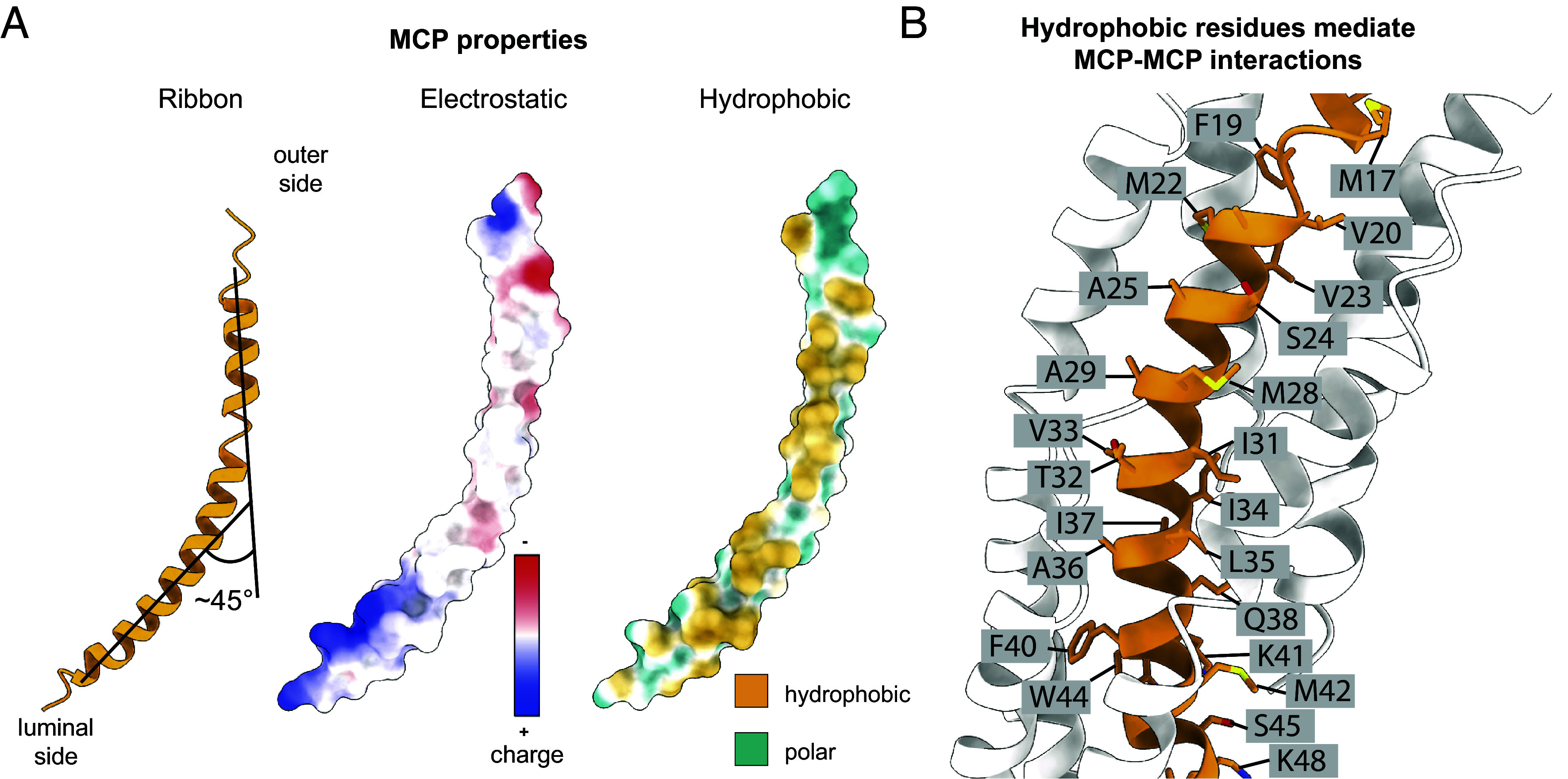
Structural characteristics of the MDAΦ MCP. (*A*) Side view of the MCP in ribbon depiction, electrostatic surface depiction, and hydrophobic surface depiction. (*B*) Side chains in the central region of the MCP, illustrating that central MCP residues are predominantly hydrophobic, mediating subunit–subunit interactions in the curved lattice of the phage.

Each MCP of MDAΦ adopts a bent architecture with significantly higher curvature than what has been seen in previous inovirus capsid structures, showing an angle of ~45° between the N- and the C-terminal segments of the MCP (Fig. 2*A*). The first six residues of the MCP are poorly resolved with smeared density in the cryo-EM map, suggesting flexibility of this region (Fig. 1 and *SI Appendix*, Fig. S2). This observation is in line with other class I bacteriophages such as Ff phages, where the N-terminal part of the MCP was found to be disordered in previous cryo-EM structures (15, 21, 22). Consistent with past structural data on inoviruses, the MCP of MDAΦ contains numerous hydrophobic residues in the central segment, which form an extensive hydrophobic network through tight packing with other MCP subunits around the filament axis (Fig. 2*B*).

### Comparison of Inovirus Structures.

**Fig. 3.**
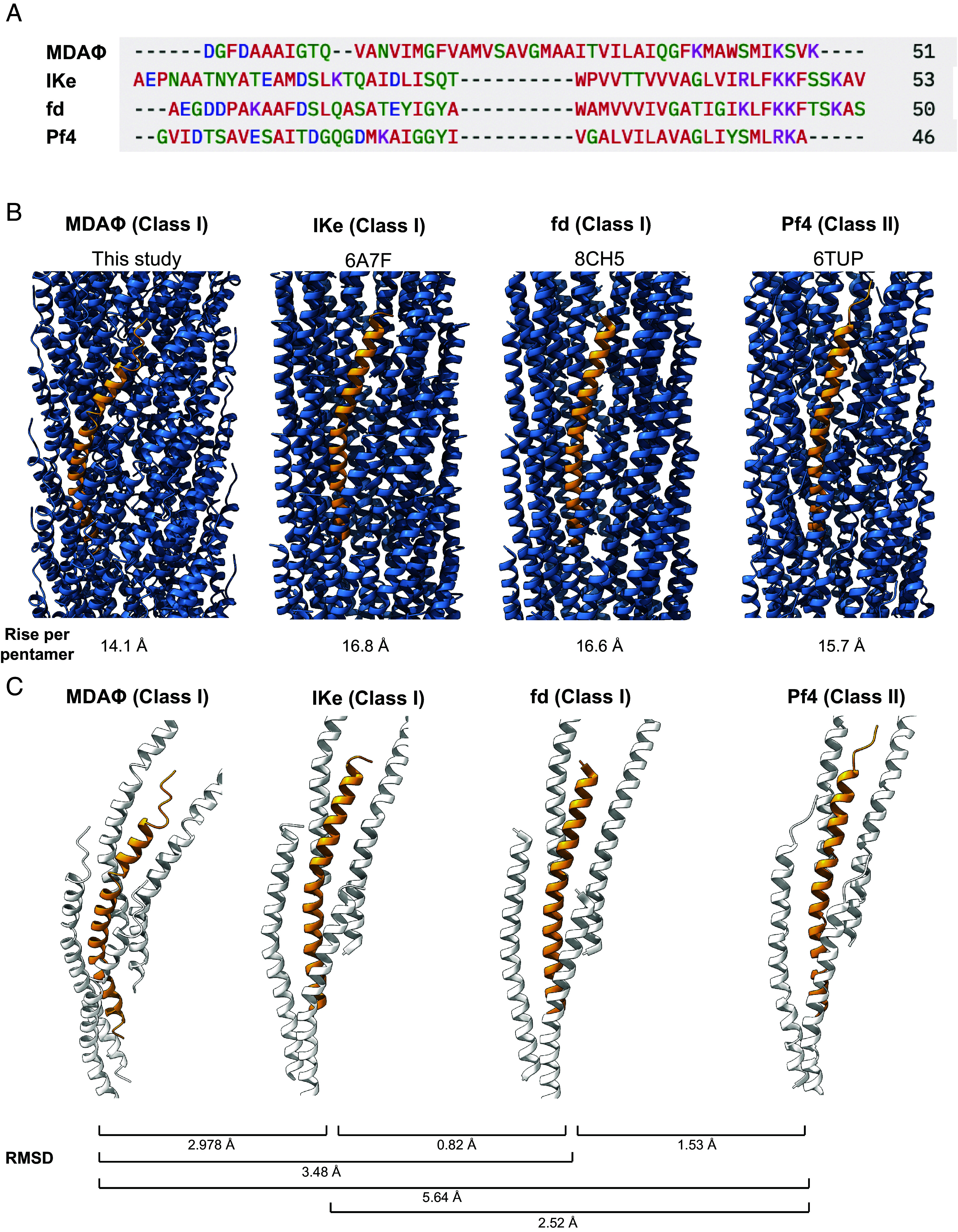
Comparison of the MDAΦ MCP with structurally characterized inoviral MCPs. (*A*) Clustal Omega sequence alignment of the mature MCP sequences of MDAΦ, IKe, fd, and Pf4. Color code: Blue: acidic residues; violet: basic residues; red: aliphatic residues; green: polar residues. (*B*) Side view of the phage MCPs, with a single MCP marked in orange. The rise is respectively given for five MCP subunits, which represents the C5-symmetric subunit in the class I bacteriophages MDAΦ, IKe, and fd. (*C*) A single MCP subunit is shown with the four closest neighboring MCP subunits. RMSD values are comparing single MCPs.

The helical parameters of the MDAΦ capsid (rise: 14.1 Å, right-handed rotation 42.3°) are markedly different from other C5-symmetric class I phages such as the Ff group of phages (fd phage rise 16.6 Å, fd phage rotation 36.4° to 37.5°) and IKe (rise 16.8 Å, rotation 38.5°), with a lower rise resulting in a comparably higher number of MCP proteins in any given capsid segment. The helical rise of the pentameric, C5-symmetric MDAΦ subunit is also smaller compared to the rise of a comparable, five-MCP subunit of the C1-symmetric class II phage Pf4 (rise of 15.7 Å for Pf4 pseudopentameric unit versus 14.1 Å for MDAΦ pentameric unit, Fig. 3).

When we compared previously solved inoviral MCP structures, we found that all inoviral MCPs are broadly similar in overall architecture and arrangement, despite large sequence diversity in different classes of inoviruses (Fig. 3). Previously solved inoviral structures show a parallel arrangement of the MCPs along the filament axis which result in largely similar intersubunit interactions. The MDAΦ MCP, on the other hand, possesses a notably more curved subunit with a higher tilt relative to the filament axis compared with previously solved phage MCPs (Figs. 2 and 3). Compared to other class I phages, which contain a highly ordered α-helical segment terminated by a helix-breaking proline residue near the N-terminus of the MCP (15, 22, 23, 25), flexibility in the MDAΦ MCP continuously increases toward the N-terminus as deduced by local resolution measurements, with the first six residues of the MCP not well resolved (*SI Appendix*, Fig. S2). On the whole, these characteristics suggest that MDAΦ represents a structurally divergent filamentous inovirus.

Little is known about the other protein components of MDAΦ, including those that form the tip proteins terminating the capsid tube and mediating receptor binding. ORF6 (NMA1797) of MDAΦ has been implicated as an adhesion protein akin to G3P in Ff phages (21). It is noteworthy that, using AlphaFold3 modeling (26), we were able to model a putative complex containing five subunits of ORF6, five subunits of ORF7 (NMA1798), together with the MCP (ORF4; NMA1795) (*SI Appendix*, Fig. S4). The predicted complex has a highly similar arrangement to a previously published cryo-EM structure of the f1 phage pointy tip (*SI Appendix*, Fig. S4) (21), suggesting that the tip architecture of MDAΦ may be similar to other class I phages.

### Binding of MCP to the Genome.

**Fig. 4.**
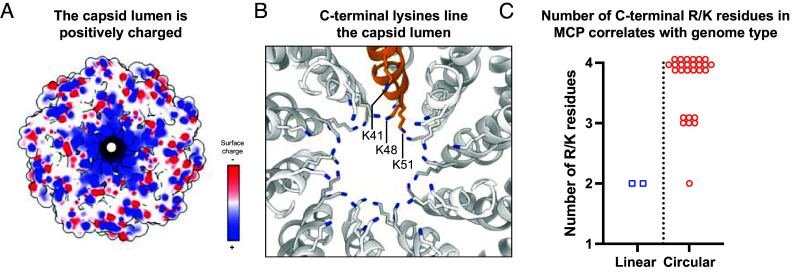
The C-terminus of the MCP coordinates the luminal DNA. (*A*) Electrostatic surface depiction of a section of the MDA phage showing positive charge within the phage lumen (blue = positively charged, red = negatively charged). (*B*) Three C-terminal lysine residues extend toward the phage genome. (*C*) Number of arginine (R) or lysine (K) residues in the C termini of the MCPs from experimentally sequenced inoviral genomes, annotated and reported by Roux et al. (18).

The C-terminus of the MDAΦ MCP, which is exposed to the phage lumen, contains three lysine residues with side chains extending toward the phage genome (Fig. 4 and *SI Appendix*, Fig. S3). Such positively charged amino acid residues are commonly found at the C termini of inoviral MCPs, which compensate the negative charge of the ssDNA genome running along the inside of the phage filament (15, 21–23). Two types of DNA genomes spanning the length of the filamentous phage capsid have been described: circular ssDNA and linear ssDNA. For circular ssDNA genomes, the genome loops back on itself, which requires the capsid to compensate twice as much charge compared to linear ssDNA, per unit length of the phage. This, in turn, requires additional positive charges in the C-terminus of each MCP for genome encapsidation.

In previous inoviral MCP structures, either four positively charged residues coordinating a circular ssDNA (Ff and Ike phages) (15, 21–23) or two positively charged residues coordinating a linear ssDNA (Pf4) were observed (14). Density for the ssDNA genome was previously uninterpretable for the class I f1, fd, and IKe phages (15, 21, 23) as well as for the MDA phage in this study (*SI Appendix*, Fig. S4), while for the class II inovirus Pf4, density consistent with a single linear ssDNA was observed (14). To assess whether the total number of C-terminal positively charged MCP residues is predictive for the arrangement of the inoviral DNA genome, we inspected available inoviral reference genomes that had previously been mined and collated in a different study (18). We only considered reference genomes where the genome type had been previously determined experimentally to be linear or circular through direct sequencing of the phage DNA. Indeed, we found that inoviruses with genomes annotated as circular almost exclusively had either 3 or 4 basic residues in the C-terminus of the MCP, while linear inoviruses had 2 basic residues in the C-terminus of their MCP (Fig. 4). Out of the 28 experimentally sequenced phage genomes, there was only a single exception, the inovirus Pf3, the genome of which was found to be circular, although its MCP contains only two basic residues. The MDAΦ genome was previously sequenced and found to be circular (27), and indeed this empirical classification scheme would place MDAΦ as a filamentous bacteriophage with a circular ssDNA, in line with the published sequencing data. The phage particle length, which was previously shown to be 1,200 nm (27), is also consistent with a circular single-stranded genome given the genome size of 8 kb, and would be incompatible with a linear ssDNA arrangement.

### The MDAΦ MCP Exposes Hydrophobic Residues on the Phage Surface.

**Fig. 5.**
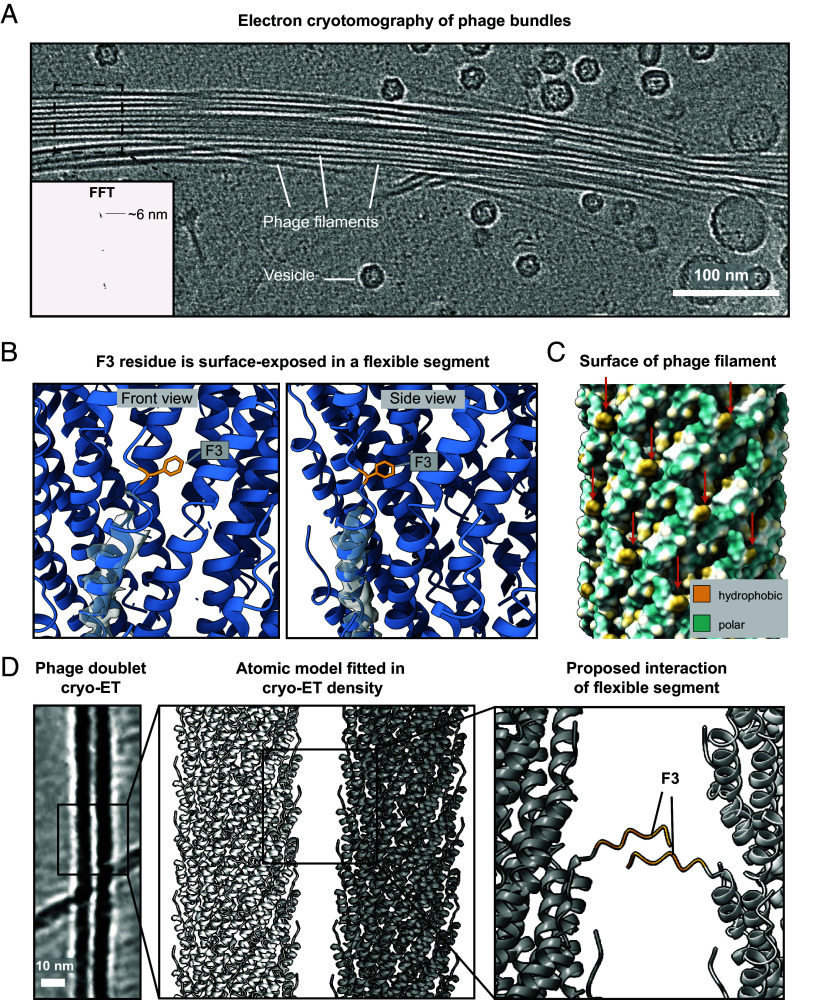
Structural analysis of phage–phage interactions. (*A*) Cryo-ET slice depicting a bundle of MDA phages. *Inset*: Power spectrum of an image segment of the bundle depicting a range of phage–phage lateral spacings centered around ~6 nm. The section used to produce the power spectrum is shown as a dashed box. (*B*) An N-terminal segment of the MCP containing the F3 residue (marked in orange) is surface-exposed. The cryo-EM density (gray, transparent) shows that the N-terminal segment containing the F3 residue is poorly resolved and likely flexible. (*C*) Hydrophobic surface depiction of the capsid (orange arrows indicate hydrophobic patches caused by F3). (*D*) *Left*: Cryo-ET slice of two phages interacting with each other laterally (in a “phage doublet”). *Middle*: Fit of the atomic model of the MDA phage capsid into the tomographic density. *Right*: Modeling of the flexible N-terminal residues (residues 1–6; orange) reveals putative interactions between N-terminal segments of neighboring phages. Since bundling occurs spontaneously in MDAΦ, without crowding agents, this provides a structural rationale to phage bundling.

Previous studies have suggested that MDAΦ filaments promote colonization of epithelial cells by promoting cohesion between bacterial cell layers (17). Virulence of *N. meningitidis* depends on the invasion of the bloodstream, which requires biofilm formation on the epithelium. Hence, how phages promote biofilm formation is of significant biomedical relevance. In our single-particle dataset, we saw several instances of MDA forming bundles, which were not observed in our previous studies on the other filamentous phages Pf4 (14) and fd (15) (*SI Appendix*, Fig. S5). Bundling of extracellular fibers is often a key mechanism of bacterial biofilm formation (28–30); therefore, understanding how filament–filament interactions are formed is important to understand mechanistic details of MDA phage fiber function. To probe this further, we imaged bundles of MDA phages present in our sample using cryo-ET, which showed a dense arrangement of phages stacked along their long axes (Fig. 5*A* and *SI Appendix*, Fig. S6). A Fourier analysis of the spacing of phages in our data reveals a weak equatorial repeat, orthogonal to the long axis of the bundle, which is close to the diameter of individual phage particles. This repeat agrees with the packing of the phages observed inside the bundles (Fig. 5*A*, box), with longitudinal alignment of the phages. These observations show that tight phage–phage interactions are present in the bundle, but variations in phage–phage spacings are possible.

To ascertain how bundling is mediated at the molecular level, we inspected our atomic model of the MDAΦ MCP and noticed that the N-terminal residues 1–6 form a surface-exposed segment with a prominent hydrophobic phenylalanine residue (F3) (Fig. 5*B*) in the poorly resolved, flexible N-terminus of the protein, which is followed by further hydrophobic residues 5–9 (sequence AAAI). Since this short segment containing the F3 residues does not appear to be rigidly constrained by the α-helicity of the MCP, we reasoned it may extend outside the phage into the external medium, where it could mediate binding to other phages to support bundle formation due to avidity effects. Due to the repetitive nature of the MCP (C5 symmetry; rise 14.1 Å, rotation per subunit 42.3°), the F3 residue results in the formation of repetitive hydrophobic patches on the phage outer surface (Fig. 5 *B* and *C*) that are ideally positioned to promote phage–phage interactions, i.e. promote bundling of phages with each other. To probe this hypothesis, we fitted our atomic model into the cryo-ET density of two phages interacting with each other in a doublet (Fig. 5*D*). Our analysis shows that while the phages within the doublet density are spaced too far to interact through their ordered capsids, they are sufficiently close for their flexible N-terminal segments to interact (Fig. 5*D*). While these interactions between the flexible segments cannot be trivially resolved through structural methods, they provide a plausible rationale for the presence of the highly hydrophobic F3 residue in this solvent-exposed flexible segment, not seen in other structurally characterized inoviruses (Fig. 3*A*). The high level of symmetry of the MDAΦ capsid moreover means that interaction through this segment results in high avidity.

**Fig. 6.**
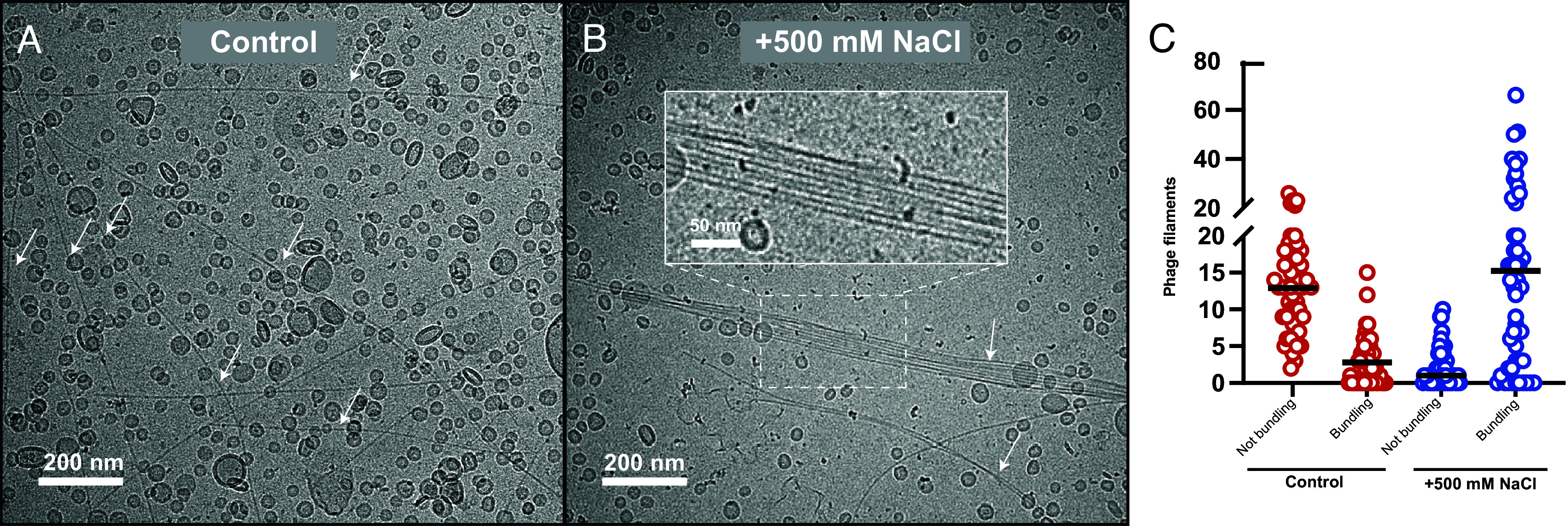
Experimental support for hydrophobicity-driven phage bundling. (*A* and *B*) Representative micrographs of the phage sample after the addition of 1:10 phosphate-buffered saline (PBS) or 1:10 5 M NaCl, followed by incubation for 18 h. Arrows point to phage filaments. (*Inset* in *B*) Zoomed-in view of a phage bundle. (*C*) Quantification of unbundled (single) or bundled phages in the two specimens in 50 randomly chosen micrographs. In each micrograph, nonbundling and bundling phages were counted; the counted number in each of the 50 micrographs is plotted. A horizontal line is drawn at the mean. The addition of NaCl results in dramatically increased bundling, supporting a model where hydrophobic interactions mediate phage–phage bundling. It should be noted that addition of salt led to the opposite effect (namely reduced bundling) in other inoviral phages Fd, Pf1, Pf3, and PH75 in a previous study (31).

To validate our phage–phage interaction model detailed above, we turned to biochemically probing bundle formation in vitro. A previous study has shown that while the filamentous phages Pf1, Pf3, fd, and PH75 can associate into bundles at low salt concentrations, this interaction is disrupted at higher salt concentrations (31), indicating that phage–phage interactions occur through electrostatic or polar contacts. Our data for the MDA phage, however, suggests that interactions through its hydrophobic N-terminal segment of the MCP mediate bundling. If this hypothesis were correct, then higher salt concentrations should increase bundling as solvent-exposed hydrophobic segments would interact with each other in order to avoid exposure to high ionic strength. To test this, we imaged the phage specimen in an increased salt concentration of 500 mM NaCl using cryo-EM. The resulting micrographs (Fig. 6 and *SI Appendix*, Fig. S7) show a striking increase in bundling compared to the control sample, supporting the proposed model where the surface-exposed hydrophobic segment in the MCP mediates phage–phage interactions.

## Discussion

Why most *N. meningitidis* populations remain commensal while others become invasive has been the subject of intensive research (1, 6). A common factor in invasive populations is the expression of MDAΦ genes, resulting in phage production (8, 9), facilitating survival on the nasopharynx epithelium (17), which is a prerequisite for invasion and virulence. Our structural data on the biomedically relevant MDA phage show that MDAΦ is a class I, C5-symmetric bacteriophage with dense MCP packing. Although only a handful in number, previous high-resolution cryo-EM structures of inoviral phages allow us to perform a detailed comparison between various phages. The α-helix of the MCP of MDAΦ is unusually curved compared to the MCPs of other structurally described class I and II inoviral phages (Fig. 3). Interestingly, our comparisons show that fd, another class I bacteriophage, is architecturally closer to the class II bacteriophage Pf4 than to MDAΦ, suggesting that this traditional classification based on the symmetry estimated in fiber diffraction experiments is not fully indicative of structural similarity of phage MCPs. Moreover, given that only model filamentous bacteriophage structures have been described previously, our structure also suggests that a significant structural diversity of filamentous phages likely exists that is yet to be uncovered, previously hinted to by bioinformatic studies (18). Indeed, previous studies have suggested that sequence conservation does not necessarily translate to conservation of quaternary architecture, which can be influenced by a change in only few amino acid residues in the sequence (32–35), further underlining this point.

In this study, we found a correlation between the number of positively charged residues at the C-terminus and the experimentally reported genome arrangement of the phage. Our analysis shows that almost all bacteriophages with circular ssDNA have MCPs with 3 or 4 C-terminal lysine or arginine residues, and phages with linear ssDNA–although less common–have 2 basic residues at the C-terminus of the MCP. This fairly simple scheme allows classification of hitherto unknown phages into respective categories without the need for detailed molecular analyses or high-resolution structure determination. The only exception to our proposed scheme was the phage Pf3, which according to this criterion would be predicted to have a linear ssDNA genome. The Pf3 MCP is similar at the sequence level to the Pf4 MCP, which was confirmed via structural methods to bind to a linear ssDNA genome (14). It is hard to reconcile these two observations in this single exception to our proposed classification scheme without further experimentation on the Pf3 phage.

Virulence of *N. meningitidis* depends on its ability to invade the bloodstream, which requires biofilm formation on the epithelium (5). Biofilms are characterized by fiber bundles that scaffold the biofilm (29), and MDAΦ had been implicated in this process. However, mechanistic details of how MDA would fulfill this role were unclear. Our work shows that each MDAΦ MCP subunit contains an unstructured N-terminal segment that presents a hydrophobic phenylalanine residue on the phage surface. While many class I phages have disordered N-terminal tails, highly hydrophobic disordered residues are not present on the surface of other structurally characterized class I bacteriophages (15, 21–23). Due to the highly repetitive nature of the MCP on the MDA phage surface, a high-avidity interaction between neighboring phages could be produced by stacking of these N-terminal phenylalanine residues, suggesting a biochemical mechanism for phage bundling without a need for crowding agents. According to this model, increasing ionic strength should increase the hydrophobic interaction between phages, and indeed, we observe a clear increase in bundling upon addition of salt (Fig. 6 and *SI Appendix*, Fig. S7). A further interesting question to be probed is whether the bundles formed by MDAΦ are in direct contact with other biofilm components during infection, which appears plausible but has not been conclusively shown.

Notably, there is precedence for phage bundling providing a beneficial effect to bacterial survival in other species. In *P. aeruginosa* for example, the filamentous phage Pf4 is expressed during infections caused by biofilms (36). Pf4 forms bundles around the bacterial cells that prevent diffusion of bactericidal molecules to the bacterial cell surface, promoting antibiotic tolerance (14, 15). Since there are no N-terminal, disordered, hydrophobic residues in the Pf4 MCP, bundling requires a crowding agent such as alginate or hyaluronan, through an entropic effect termed depletion attraction (11). It is conceivable that physiologically relevant crowding agents such as hyaluronan could further enhance MDAΦ phage–phage interactions in a biomedical setting to support the formation of large phage bundles, although this will require experimental validation in future studies. Another beneficial effect of Pf4 phage expression at the sites of *P. aeruginosa* infection is the triggering of human antiviral immune responses, downregulating the antibacterial response, promoting bacterial survival (12, 36). It has been previously shown that MDAΦ bundling reinforces bacterial adhesion (17); whether this also triggers an antiviral response during *N. meningitidis* infection, thus aiding bacterial survival, remains to be determined. Another notable example of a filamentous phage bolstering virulence is provided by CTXΦ in *V. cholerae*, where the prophage CTX encodes the cholera toxin genes that cause the symptoms of the disease (16). The fact that inoviruses are being employed by numerous bacterial pathogens, all of which appear to exploit the properties of the phage in differing ways, suggests that symbiotic relationships between inoviruses and bacteria may be more common than previously envisaged. This use of symbiotic phages by bacteria to enhance survival is an area of biomedical research that needs urgent attention in future studies.

## Methods

### Isolation of Phage Particles.

For phage isolation, the strain Z5463, formerly designated C396, isolated from the throat of a patient with meningitis in The Gambia in 1983 (37) was used. As previously described (27), bacteria were pelleted from 400 mL of an overnight culture in BHI liquid medium (Condalab) at 37 °C with 5% CO_2_ and agitation. After filtration with a 0.45 µm filter, the supernatant was treated for 2 h at 20 °C with DNase I (25 µg/mL). Phage was precipitated by the addition of 0.5 M NaCl and 10% polyethylene glycol (PEG) 6000, and incubation overnight at 4 °C. The phage was then pelleted by centrifugation at 10,000× relative centrifugal force (rcf) for 30 min and resuspended in PBS (phosphate-buffered saline) 1X (with CaCl_2_ and MgCl_2_; Gibco). To further concentrate the phage preparation for cryo-EM, the sample was once more adjusted to 0.5 M NaCl and 10% (w/v) PEG 6000 before overnight incubation at 4 °C. Phage particles were pelleted by centrifugation at 15,000 rcf for 30 min. The resulting pellet containing phage was resuspended in PBS followed by dialysis against PBS overnight at 4 °C using 10 kDa Molecular Weight Cut-Off snakeskin dialysis membranes (ThermoFisher).

### Cryo-EM Grid Preparation.

For cryo-EM and cryo-ET grid preparation, previously described protocols were utilized (38). Briefly, 2.5 μL of the concentrated phage was pipetted onto glow discharged Quantifoil grids (Au R1.2/1.3, 400 mesh) and plunge frozen into liquid ethane using a Vitrobot Mark IV (ThermoFisher), with the chamber maintained at 100% relative humidity and 10 °C.

### Cryo-EM Image Acquisition and Processing.

Cryo-EM data were acquired in the EER format on a Titan Krios G2 microscope equipped with a Falcon 4 detector, at a nominal magnification of 96,000x using a combined dose of 41 e/Å^2^ per movie.

### Symmetry Determination and Image Processing.

EER movies were converted to TIF and motion-corrected using the RELION4 implementation of MotionCor2 (39, 40). CTF refinement was performed using CTFFIND4 (41). Phage particles were picked using Topaz (42). Estimating the helical symmetry based on indexing of layer lines observed in Fourier transforms of 2D class averages (power spectrum shown in *SI Appendix*, Fig. S2*B*) was found to be unreliable. However, a repeat with a rise of ~72 Å is visible in the class averages (*SI Appendix*, Fig. S2*A*), which we hypothesized represents a full turn of the helix. We further hypothesized that the phage capsid symmetry would likely have either a C1 or C5 rotational symmetry, consistent with previously characterized filamentous phages.

Since a defined integer number of helical subunits must form the 72 Å helical turn, this leads to a finite set of possibilities for the helical symmetry, given the size of the α-helical capsid protein. In the case of C5 symmetry, given a similar MCP length and diameter to previously solved filamentous phages, the rise of MDA filament would likely be either 4 subunits (producing a 72 Å/4 = 18 Å rise/subunit), 5 subunits (14.4 Å rise/subunit), or 6 subunits (12 Å rise/subunit). For each rise, a range of twists are theoretically possible, but C5 symmetry limits the possibilities to a range from 0° to 360°/5 = 72°. A range of twists were thus tested for each possible rise, yielding α-helical MCP density and <4 Å resolution solely for a 14.2 Å rise/subunit and 42.6° twist/subunit. Helical parameters were searched during refinements; the final helical parameters were a 14.1 Å rise/subunit and 42.3° twist/subunit with C5 symmetry. Despite our attempts to resolve the structure of the ssDNA genome using unsymmetrized, symmetrized, and masked refinements, none of the attempts yielded an interpretable density of the genome that supported atomic model building. All 3D classifications and refinements were performed in RELION4 (40, 43). A workflow for the performed image processing steps is shown in *SI Appendix*, Fig. S1. All image processing was performed in RELION, apart from the 2D class average shown in *SI Appendix*, Fig. S2 *A* and *B*, which was produced in cryoSPARC (44), which resulted in slightly improved visibility of layer lines in Fourier transforms.

### Atomic Model Building.

An atomic model of the MDA phage capsid was built with Coot using a structural prediction by AlphaFold2 as a starting model. Multiple chains were built to account for subunit–subunit interactions. Refinement was initially performed in PHENIX (45) and then in Servalcat (46) using Servalcat’s helical refinement pipeline. Initially, the “servalcat util symmodel” command was used to construct a helical assembly from a single subunit, enabling manual model building. Subsequent refinements against the cryo-EM density were then performed using the specific helical parameters according to the software manufacturer’s instruction (47). The final refinement was performed in Servalcat. Map and model statistics are shown in *SI Appendix*, Table S1. The rotation matrices for the assembly deposited to the PDB were generated using the “servalcat util helical_biomt” function.

### Cryo-ET Imaging.

Cryo-ET data were acquired on a Titan Krios G4 microscope equipped with a Falcon 4i detector and SelectrisX energy filter, at a nominal magnification of 53,000x and pixel size of 2.39 Å. Tilt series were acquired from −60° to +60° with 3° increments using a dose-symmetric tilt scheme in SerialEM (48) and a total dose of 120 e^−^/Å^2^ over the tilt series at −5 to −7 µm nominal defoci. Tomograms were reconstructed using the etomo package implemented in IMOD (49) using patch tracking and SIRT, or using SART as implemented in AreTomo (50). The cryoCARE software package was used for denoising of tomograms (51).

### Phage MCP Sequence Alignment and Genome Analysis.

Sequence alignment of MCPs was performed using Clustal Omega as implemented on the EBI web server (52). For the analysis of basic C-terminal residues, amino acid sequences of the MCPs in experimentally sequenced, reference genomes as reported by Roux et al. (18) were used, and the occurrence of lysine or arginine residues in the C-terminal 15 residues was assessed. A comprehensive list of analyzed genomes and MCPs is given in *SI Appendix*, Table S2.

### Quantification of Phage Bundling.

To determine the effect of salt concentration on phage bundling, freshly precipitated phage was incubated with either 1:10 5 M NaCl or the equivalent volume of PBS for 18 h. The resulting specimens were plunge-frozen on Quantifoil R2/2 Au 200 mesh grids. ~350 micrographs were acquired for each condition at 36,000x nominal magnification and −10 µm defocus on a ThermoFisher Scientific Glacios 200 kV electron cryomicroscope equipped with a Falcon 3 direct electron detector. For each condition, 50 images were randomly chosen using the “shuf -n” function in Unix. In the resulting images, a phage was considered to be bundling if it was laterally associated with at least one other phage over at least half its length. The individual quantification values are plotted in Fig. 6.

### Data Visualization.

Atomic models were visualized in ChimeraX (53). FSC plots were created in MATLAB (R2022b), and the genome type plot as well as quantification graph for phage bundling were created GraphPad Prism and subsequently manually adjusted. Micrographs were filtered in Fiji (54) and displayed using Fiji or IMOD (49). Structural predictions were performed using the AlphaFold3 web server (26). Cryo-ET data were visualized using IMOD.

## Supplementary Material

Appendix 01 (PDF)

Movie S1. **Cryo-EM structure of the MDAΦ capsid**. Cryo-EM density and ribbon depiction of the atomic model of the MDAΦ capsid are shown.

Movie S2. **Electron cryotomography of MDAΦ bundles**.Shown are sequential Z-slices of a tomogram of an MDAΦ bundle.

## Data Availability

The cryo-EM atomic model and density of the MDA phage capsid have been deposited at the Protein Data Bank (PDB) under accession ID (9QG9, 55), and at the Electron Microscopy Data Bank (EMDB) under accession code (EMD-53129, 56). All other data are included in the manuscript and/or supporting information.

## References

[1] B. B. Mook-Kanamori, M. Geldhoff, T. van der Poll, D. van de Beek, Pathogenesis and pathophysiology of pneumococcal meningitis. Clin. Microbiol. Rev. **24**, 557–591 (2011). 21734248 10.1128/CMR.00008-11 PMC3131058

[2] H. S. Seifert, Location, location, location-commensalism, damage and evolution of the pathogenic *Neisseria*. J. Mol. Biol. **431**, 3010–3014 (2019). 30986425 10.1016/j.jmb.2019.04.007

[3] M. Pizza, R. Rappuoli, *Neisseria meningitidis*: Pathogenesis and immunity. Curr. Opin. Microbiol. **23**, 68–72 (2015). 25461575 10.1016/j.mib.2014.11.006

[4] X. Nassif, M. So, Interaction of pathogenic *Neisseriae* with nonphagocytic cells. Clin. Microbiol. Rev. **8**, 376–388 (1995). 7553571 10.1128/cmr.8.3.376 PMC174630

[5] M. Coureuil , Molecular interactions between *Neisseria meningitidis* and its human host. Cell Microbiol. **21**, e13063 (2019). 31167044 10.1111/cmi.13063 PMC6899865

[6] A. J. Merz, M. So, Interactions of pathogenic *Neisseriae* with epithelial cell membranes. Annu. Rev. Cell Dev. Biol. **16**, 423–457 (2000). 11031243 10.1146/annurev.cellbio.16.1.423

[7] D. J. Hill, N. J. Griffiths, E. Borodina, M. Virji, Cellular and molecular biology of *Neisseria meningitidis* colonization and invasive disease. Clin. Sci. (Lond.). **118**, 547–564 (2010). 20132098 10.1042/CS20090513 PMC2830671

[8] E. Bille , A chromosomally integrated bacteriophage in invasive meningococci. J. Exp. Med. **201**, 1905–1913 (2005). 15967821 10.1084/jem.20050112 PMC2212043

[9] E. Bille , Association of a bacteriophage with meningococcal disease in young adults. PLoS ONE **3**, e3885 (2008). 19065260 10.1371/journal.pone.0003885 PMC2587699

[10] D. Marvin, M. Symmons, S. Straus, Structure and assembly of filamentous bacteriophages. Prog. Biophys. Mol. Biol. **114**, 80–122 (2014). 24582831 10.1016/j.pbiomolbio.2014.02.003

[11] P. R. Secor , Filamentous bacteriophage promote biofilm assembly and function. Cell Host Microbe **18**, 549–559 (2015). 26567508 10.1016/j.chom.2015.10.013 PMC4653043

[12] E. B. Burgener , Filamentous bacteriophages are associated with chronic *Pseudomonas* lung infections and antibiotic resistance in cystic fibrosis. Sci. Transl. Med. **11**, eaau9748 (2019). 30996083 10.1126/scitranslmed.aau9748 PMC7021451

[13] J. Bondy-Denomy, A. R. Davidson, When a virus is not a parasite: The beneficial effects of prophages on bacterial fitness. J. Microbiol. **52**, 235–242 (2014). 24585054 10.1007/s12275-014-4083-3

[14] A. K. Tarafder , Phage liquid crystalline droplets form occlusive sheaths that encapsulate and protect infectious rod-shaped bacteria. Proc. Natl. Acad. Sci. U. S. A. **117**, 4724–4731 (2020). 32071243 10.1073/pnas.1917726117 PMC7060675

[15] J. Böhning , Biophysical basis of filamentous phage tactoid-mediated antibiotic tolerance in *P. aeruginosa*. Nat. Commun. **14**, 8429 (2023). 38114502 10.1038/s41467-023-44160-8 PMC10730611

[16] M. K. Waldor, J. J. Mekalanos, Lysogenic conversion by a filamentous phage encoding cholera toxin. Science **272**, 1910–1914 (1996). 8658163 10.1126/science.272.5270.1910

[17] E. Bille , A virulence-associated filamentous bacteriophage of *Neisseria meningitidis* increases host-cell colonisation. PLoS Pathog. **13**, e1006495 (2017). 28704569 10.1371/journal.ppat.1006495 PMC5526601

[18] S. Roux , Cryptic inoviruses revealed as pervasive in bacteria and archaea across Earth’s biomes. Nat. Microbiol. **4**, 1895–1906 (2019). 31332386 10.1038/s41564-019-0510-x PMC6813254

[19] D. A. Marvin, R. L. Wiseman, E. J. Wachtel, Filamentous bacterial viruses. XI. Molecular architecture of the class II (Pf1, Xf) virion. J. Mol. Biol. **82**, 121–138 (1974). 4206038 10.1016/0022-2836(74)90336-2

[20] D. A. Marvin, W. J. Pigram, R. L. Wiseman, E. J. Wachtel, F. J. Marvin, Filamentous bacterial viruses. Virion. Molecular architecture of the class I (fd, If1, IKe). J. Mol. Biol. **88**, 581–598 (1974). 4449121 10.1016/0022-2836(74)90409-4

[21] R. Conners , Cryo-electron microscopy of the F1 filamentous phage reveals insights into viral infection and assembly. Nat. Commun. **14**, 2724 (2023). 37169795 10.1038/s41467-023-37915-w PMC10175506

[22] Q. Jia, Y. Xiang, Cryo-EM structure of a bacteriophage M13 mini variant. Nat. Commun. **14**, 5421 (2023). 37669979 10.1038/s41467-023-41151-7 PMC10480500

[23] J. Xu, N. Dayan, A. Goldbourt, Y. Xiang, Cryo-electron microscopy structure of the filamentous bacteriophage IKe. Proc. Natl. Acad. Sci. U. S. A. **116**, 5493-5498 (2019). 30819888 10.1073/pnas.1811929116 PMC6431161

[24] S. He, S. H. Scheres, Helical reconstruction in RELION. J. Struct. Biol. **198**, 163–176 (2017). 28193500 10.1016/j.jsb.2017.02.003 PMC5479445

[25] D. Marvin, L. Welsh, M. Symmons, W. Scott, S. Straus, Molecular structure of fd (f1, M13) filamentous bacteriophage refined with respect to X-ray fibre diffraction and solid-state NMR data supports specific models of phage assembly at the bacterial membrane. J. Mol. Biol. **355**, 294–309 (2006). 16300790 10.1016/j.jmb.2005.10.048

[26] J. Abramson , Accurate structure prediction of biomolecular interactions with AlphaFold 3. Nature **630**, 493–500 (2024). 38718835 10.1038/s41586-024-07487-w PMC11168924

[27] J. Meyer , Characterization of MDAΦ, a temperate filamentous bacteriophage of *Neisseria meningitidis*. MicrobiOL. Read. Engl. **162**, 268–282 (2016). 10.1099/mic.0.000215 26602366

[28] J. Böhning , Donor-strand exchange drives assembly of the TasA scaffold in *Bacillus subtilis* biofilms. Nat. Commun. **13**, 1–12 (2022). 36400765 10.1038/s41467-022-34700-z PMC9674648

[29] J. Böhning, A. K. Tarafder, T. A. M. Bharat, The role of filamentous matrix molecules in shaping the architecture and emergent properties of bacterial biofilms. Biochem. J. **481**, 245–263 (2024). 38358118 10.1042/BCJ20210301 PMC10903470

[30] A. Taglialegna, I. Lasa, J. Valle, Amyloid structures as biofilm matrix scaffolds. J. Bacteriol. **198**, 2579–2588 (2016). 27185827 10.1128/JB.00122-16 PMC5019065

[31] S. A. Overman, D. M. Kristensen, P. Bondre, B. Hewitt, G. J. Thomas Jr., Effects of virion and salt concentrations on the raman signatures of filamentous phages fd, Pf1, Pf3, and PH75. Biochemistry **43**, 13129–13136 (2004). 15476406 10.1021/bi0485023

[32] E. H. Egelman , Structural plasticity of helical nanotubes based on coiled-coil assemblies. Structure **23**, 280–289 (2015). 25620001 10.1016/j.str.2014.12.008 PMC4318749

[33] V. E. Galkin , Divergence of quaternary structures among bacterial flagellar filaments. Science **320**, 382–385 (2008). 18420936 10.1126/science.1155307

[34] M. L. Coates, Hemoglobin function in the vertebrates: An evolutionary model. J. Mol. Evol. **6**, 285–307 (1975). 1543 10.1007/BF01794636

[35] F. K. Schur , Structure of the immature HIV-1 capsid in intact virus particles at 8.8 Å resolution. Nature **517**, 505–508 (2015). 25363765 10.1038/nature13838

[36] J. M. Sweere , Bacteriophage trigger antiviral immunity and prevent clearance of bacterial infection. Science **363**, eaat9691 (2019). 30923196 10.1126/science.aat9691 PMC6656896

[37] M. Achtman , Purification and characterization of eight class 5 outer membrane protein variants from a clone of *Neisseria meningitidis* serogroup A. J. Exp. Med. **168**, 507–525 (1988). 2457646 10.1084/jem.168.2.507 PMC2189001

[38] H. Ochner , Structure of the *Pseudomonas aeruginosa* PAO1 type IV pilus. PLoS Pathog. **20**, e1012773 (2024). 39666767 10.1371/journal.ppat.1012773 PMC11670995

[39] S. Q. Zheng , Motioncor2: Anisotropic correction of beam-induced motion for improved cryo-electron microscopy. Nat. Methods **14**, 331–332 (2017). 28250466 10.1038/nmeth.4193 PMC5494038

[40] S. H. Scheres, RELION: Implementation of a Bayesian approach to cryo-EM structure determination. J. Struct. Biol. **180**, 519–530 (2012). 23000701 10.1016/j.jsb.2012.09.006 PMC3690530

[41] A. Rohou, N. Grigorieff, CTFFIND4: Fast and accurate defocus estimation from electron micrographs. J. Struct. Biol. **192**, 216–221 (2015). 26278980 10.1016/j.jsb.2015.08.008 PMC6760662

[42] T. Bepler , Positive-unlabeled convolutional neural networks for particle picking in cryo-electron micrographs. Nat. Methods **16**, 1153–1160 (2019). 31591578 10.1038/s41592-019-0575-8 PMC6858545

[43] D. Kimanius, L. Dong, G. Sharov, T. Nakane, S. H. W. Scheres, New tools for automated cryo-EM single-particle analysis in RELION-4.0. Biochem. J. **478**, 4169–4185 (2021). 34783343 10.1042/BCJ20210708 PMC8786306

[44] A. Punjani, J. L. Rubinstein, D. J. Fleet, M. A. Brubaker, cryoSPARC: algorithms for rapid unsupervised cryo-EM structure determination. Nat. Methods **14**, 290–296 (2017). 28165473 10.1038/nmeth.4169

[45] P. D. Adams , PHENIX: A comprehensive Python-based system for macromolecular structure solution. Acta Crystallogr. Sect. D Biol. Crystallogr. **66**, 213–221 (2010). 20124702 10.1107/S0907444909052925 PMC2815670

[46] K. Yamashita, C. M. Palmer, T. Burnley, G. N. Murshudov, Cryo-EM single-particle structure refinement and map calculation using Servalcat. Acta Crystallogr. D Struct. Biol. **77**, 1282–1291 (2021). 34605431 10.1107/S2059798321009475 PMC8489229

[47] K. Yamashita, Refinement of amyloid-β 42 structure (2025). https://servalcat.readthedocs.io/en/latest/spa_examples/ab42.html. Accessed 10 January 2025.

[48] D. N. Mastronarde, Automated electron microscope tomography using robust prediction of specimen movements. J. Struct. Biol. **152**, 36–51 (2005). 16182563 10.1016/j.jsb.2005.07.007

[49] J. R. Kremer, D. N. Mastronarde, J. R. McIntosh, Computer visualization of three-dimensional image data using IMOD. J. Struct. Biol. **116**, 71–76 (1996). 8742726 10.1006/jsbi.1996.0013

[50] S. Zheng , Aretomo: An integrated software package for automated marker-free, motion-corrected cryo-electron tomographic alignment and reconstruction. J. Struct. Biol. X. **6**, 100068 (2022). 35601683 10.1016/j.yjsbx.2022.100068 PMC9117686

[51] T.-O. Buchholz, M. Jordan, G. Pigino, F. Jug, "Cryo-CARE: Content-aware image restoration for cryo-transmission electron microscopy data" in 2019 IEEE 16th International Symposium on Biomedical Imaging (ISBI, 2019), pp 502-506.

[52] F. Madeira , The EMBL-EBI job dispatcher sequence analysis tools framework in 2024. Nucleic Acids Res. **52**, W521–W525 (2024). 38597606 10.1093/nar/gkae241 PMC11223882

[53] E. C. Meng , UCSF chimeraX: Tools for structure building and analysis. Protein Sci. **32**, e4792 (2023). 37774136 10.1002/pro.4792 PMC10588335

[54] J. Schindelin , Fiji: An open-source platform for biological-image analysis. Nat. Methods **9**, 676–682 (2012). 22743772 10.1038/nmeth.2019 PMC3855844

[55] J. Boehning, T. A. M. Bharat, MDA phage capsid. Protein Data Bank. https://www.rcsb.org/structure/9QG9. Deposited 13 March 2025.

[56] J. Boehning, T. A. M. Bharat, MDA phage capsid. Electron Microscopy Data Bank. https://www.ebi.ac.uk/emdb/EMD-53129. Deposited 13 March 2025.

