# Supplementary material for: Structure of the virulence-associated Neisseria meningitidis filamentous bacteriophage MDAΦ
Source: Proc Natl Acad Sci U S A. 2025 Jun 20;122(25):e2420157122. doi: 10.1073/pnas.2420157122 (PMC12207478; doi:10.1073/pnas.2420157122)
Supplement: Supplementary file 1 — Appendix 01 (PDF) [file pnas.2420157122.sapp.pdf]

PNAS

Supplementary Information for

Title

Structure of the virulence-associated *Neisseria meningitidis* filamentous bacteriophage MDAΦ

Authors

Jan Böhning[1], Miles Graham[1], Mathieu Coureuil[2], Abul K. Tarafder[1], Julie Meyer[2], Xavier Nassif[2], Emmanuelle Bille[2], Tanmay A. M. Bharat[1,*]

Affiliations

[1] Structural Studies Division, MRC Laboratory of Molecular Biology, Francis Crick Avenue, Cambridge CB2 0QH, United Kingdom

[2] INSERM U1151, CNRS UMR8253, Institut Necker-Enfants Malades, Université Paris Cité, F-75015 Paris, France

* Correspondence

Tanmay A.M. Bharat, email: tbharat@mrc-lmb.cam.ac.uk

This PDF file includes:

Figures S1 to S7
Tables S1 to S2
Legend for Movies S1 and S2
SI References

Other supplementary materials for this manuscript include:

Movie S1-2

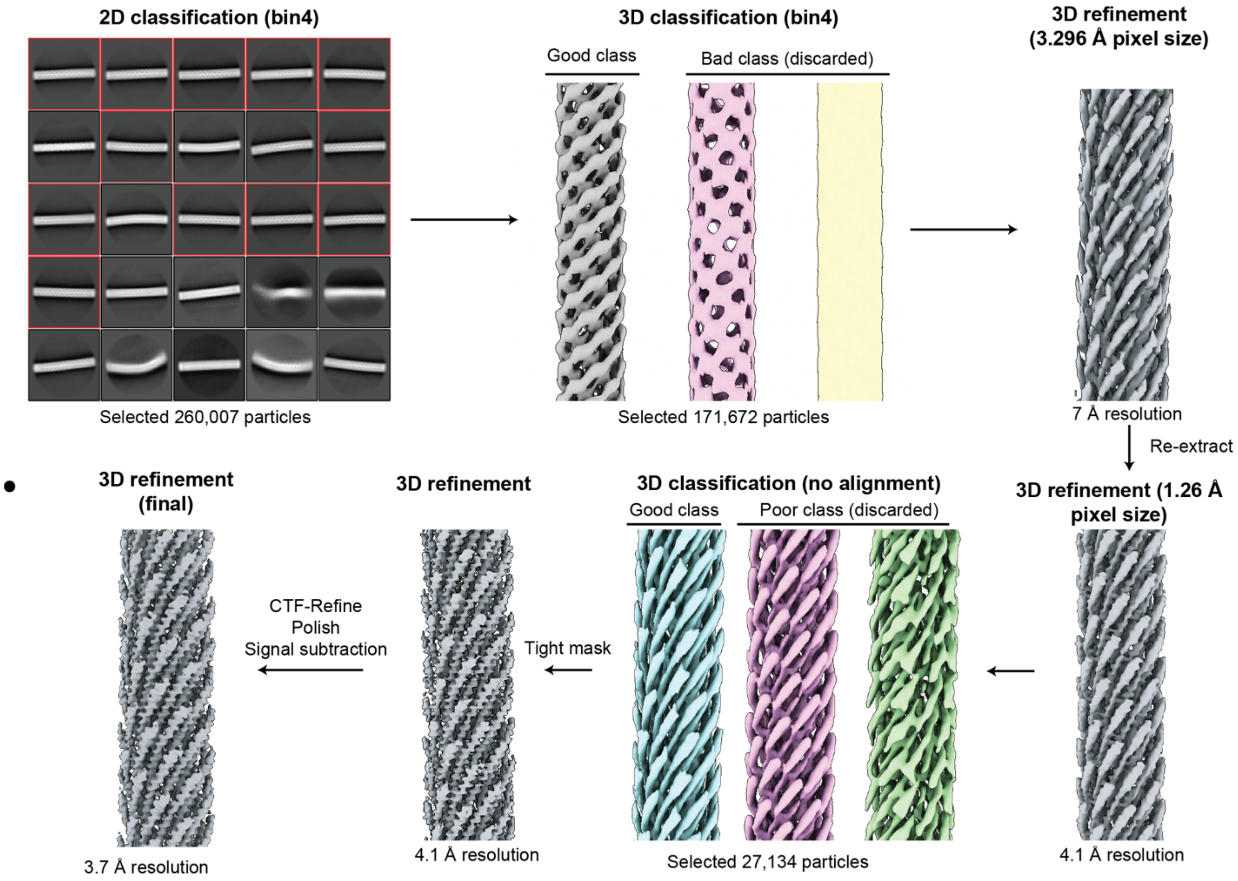

Figure S1: Cryo-EM data processing workflow.

Shown are the major classes selected, including particle numbers and further processing steps. Red squares show selected 2D classes; classes not marked were discarded.

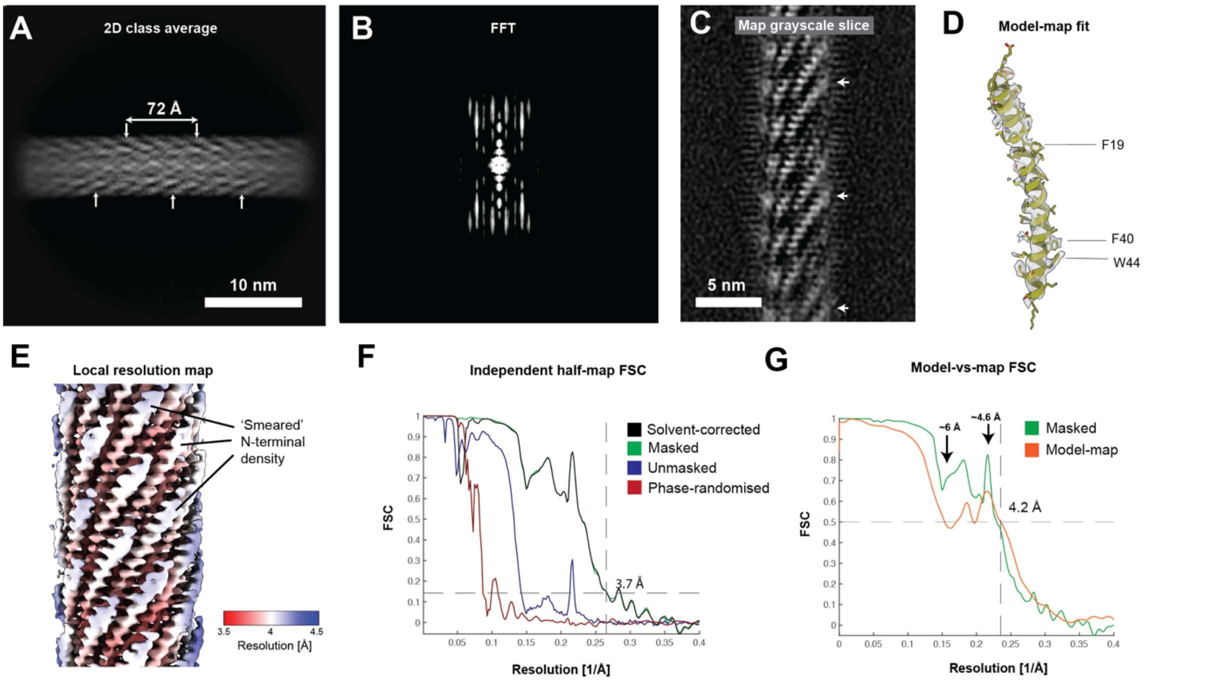

Figure S2: Cryo-EM map characteristics.

A) A 2D class average of MDA phage. A helical repeat of 72 Å visible in the 2D class average is marked. **B)** Power spectrum of the class average shown in A). **C)** Grayscale orthogonal slice of the final post-processed map. Arrows are indicating the increased disorder ('smear') towards the N-terminus within the density. **D)** Fit of the atomic model of one MCP subunit into the cryo-EM map. Aromatic side chain residues resolved in the density are marked. **E)** Cryo-EM map coloured by local resolution, scale bar shows resolution values. **F)** Resolution estimation by Fourier shell correlation (FSC) of independently aligned and averaged half-maps. Dashed line indicates the 0.143 criterion. **G)** Model-vs-map FSC as an output from Servalcat plotted with the masked gold-standard FSC curve from F). Dashed line indicates the 0.5 criterion. A dip in FSC around ~6 Å is visible in the FSC curve, likely corresponding to flexibility within the N-terminus of the MCP helix.

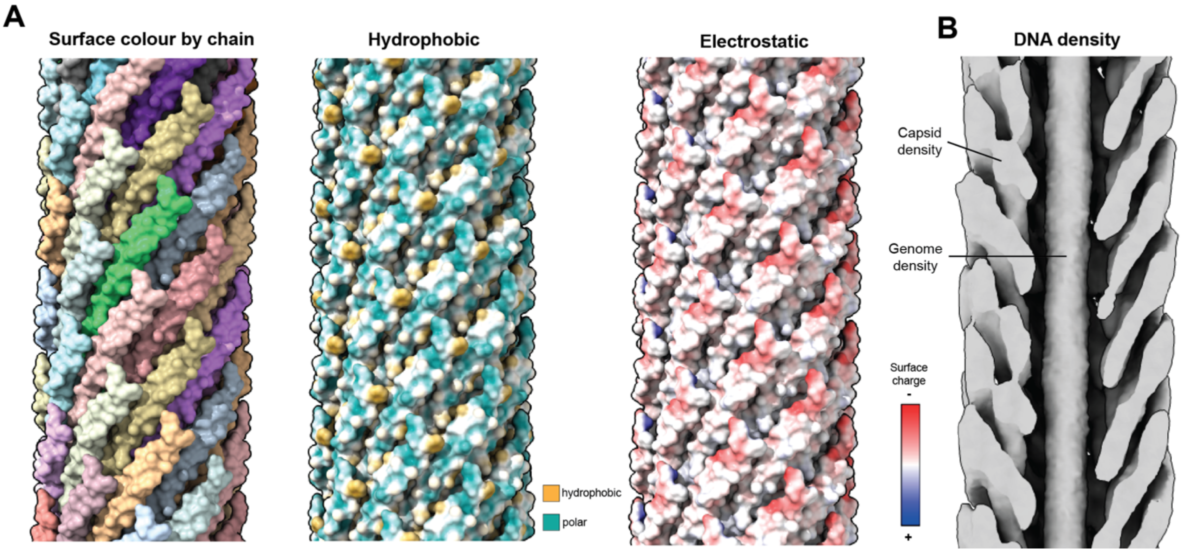

Figure S3: Surface properties of MDAΦ and genome density in the cryo-EM map.

A) Model of the MDAΦ capsid is shown, surface coloured by chain, as hydrophobic surface depiction (cyan = hydrophilic, yellow = hydrophobic) and electrostatic surface depiction (red = negatively charged, blue = positively charged). **B)** DNA density in the lumen of the MDAΦ phage, in a cryo-EM map produced with C1 (no rotational) symmetry applied, indicating that DNA features are not smeared by the C5 symmetry, because the genome appears as a featureless density in the centre of the phage capsid, consistent with other class I inoviruses.

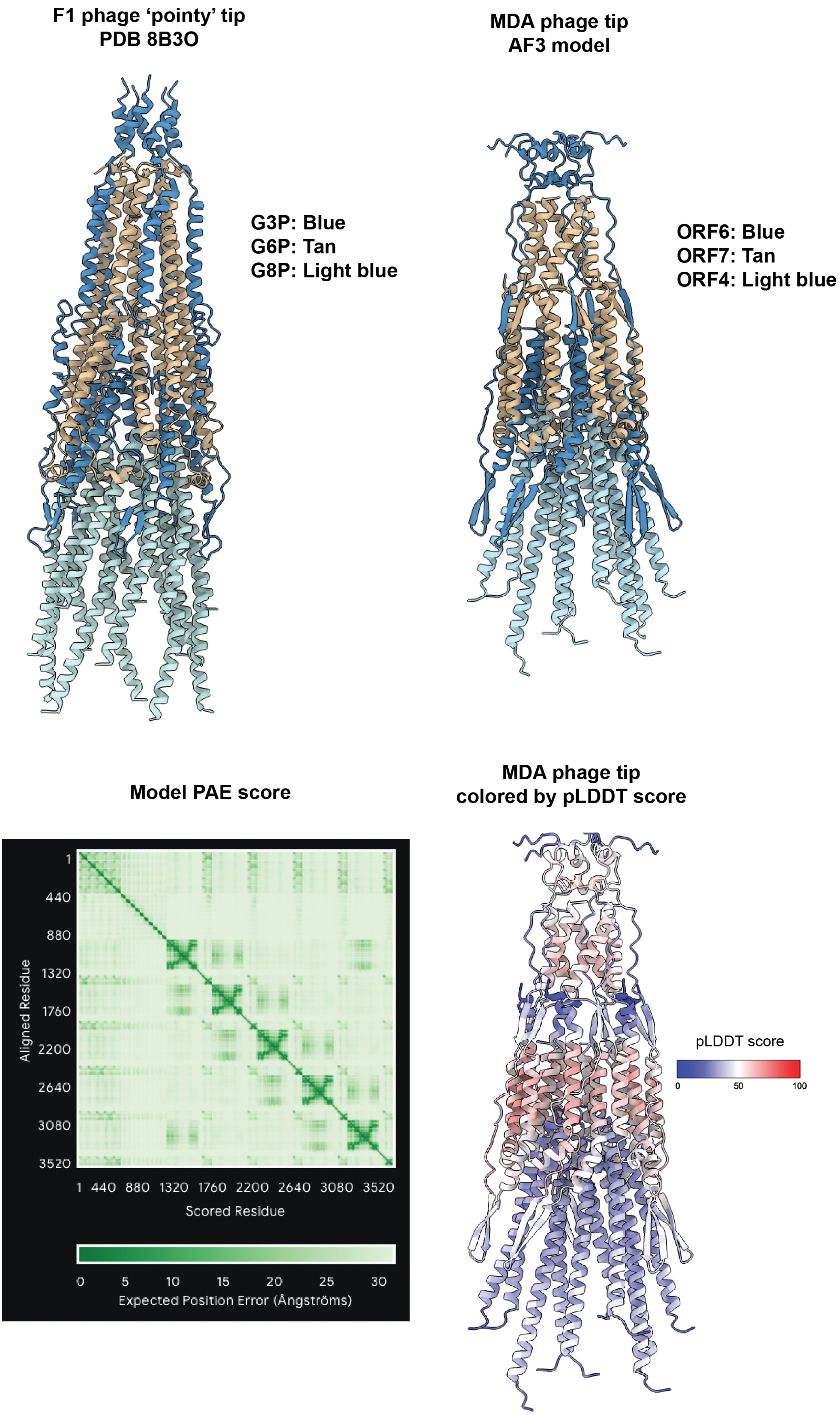
F1 phage 'pointy' tip
PDB 8B3O
G3P: Blue
G6P: Tan
G8P: Light blue
MDA phage tip
AF3 model
ORF6: Blue
ORF7: Tan
ORF4: Light blue
Model PAE score
Aligned Residue
1 440 880 1320 1760 2200 2640 3080 3520
Scored Residue
0 5 10 15 20 25 30
Expected Position Error (Ångströms)
MDA phage tip
colored by pLDDT score
pLDDT score
0 50 100

Figure S4: AlphaFold3 modelling of the MDA 'pointy' phage tip, and comparison with a cryo-EM structure of the tip of the f1 bacteriophage.

This prediction containing 10 copies of the major capsid protein ORF4, 5 copies of ORF6 and 5 copies of ORF7 is architecturally highly similar to a previously solved cryo-EM structure of the f1 filamentous phage tip (PDB 8B3O (1)). N-terminal residues 1-399 of ORF6 were excluded to enable a better structural comparison. The prediction's pTM score is 0.3 and TM score is 0.33. This proposed model will require experimental validation.

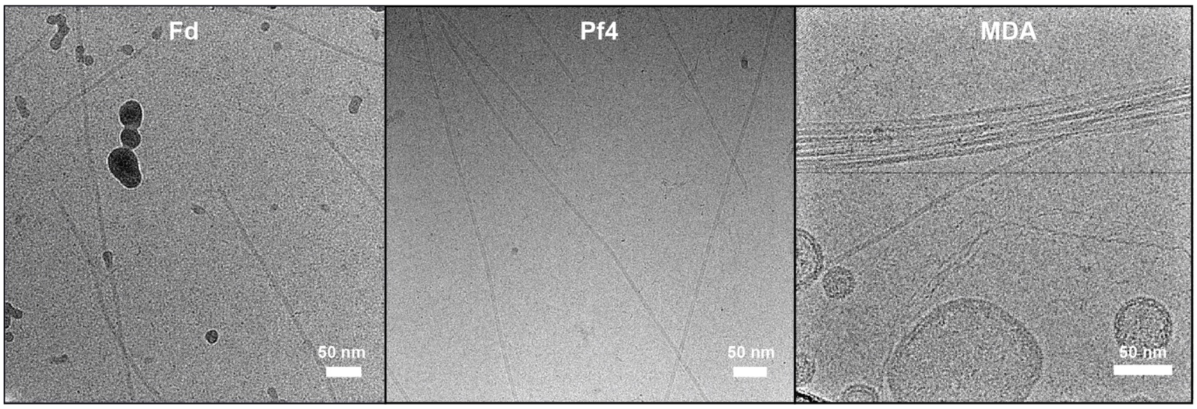

Figure S5: The formation of large phage bundles in phosphate-buffered saline (PBS) is observed for MDA phage but not for other filamentous phages.

Left and middle: example micrographs of Fd and Pf4 in PBS from single-particle cryo-EM datasets of previous studies (2, 3). Right: MDA phage filaments in PBS were found to have a propensity to assemble into large bundles.

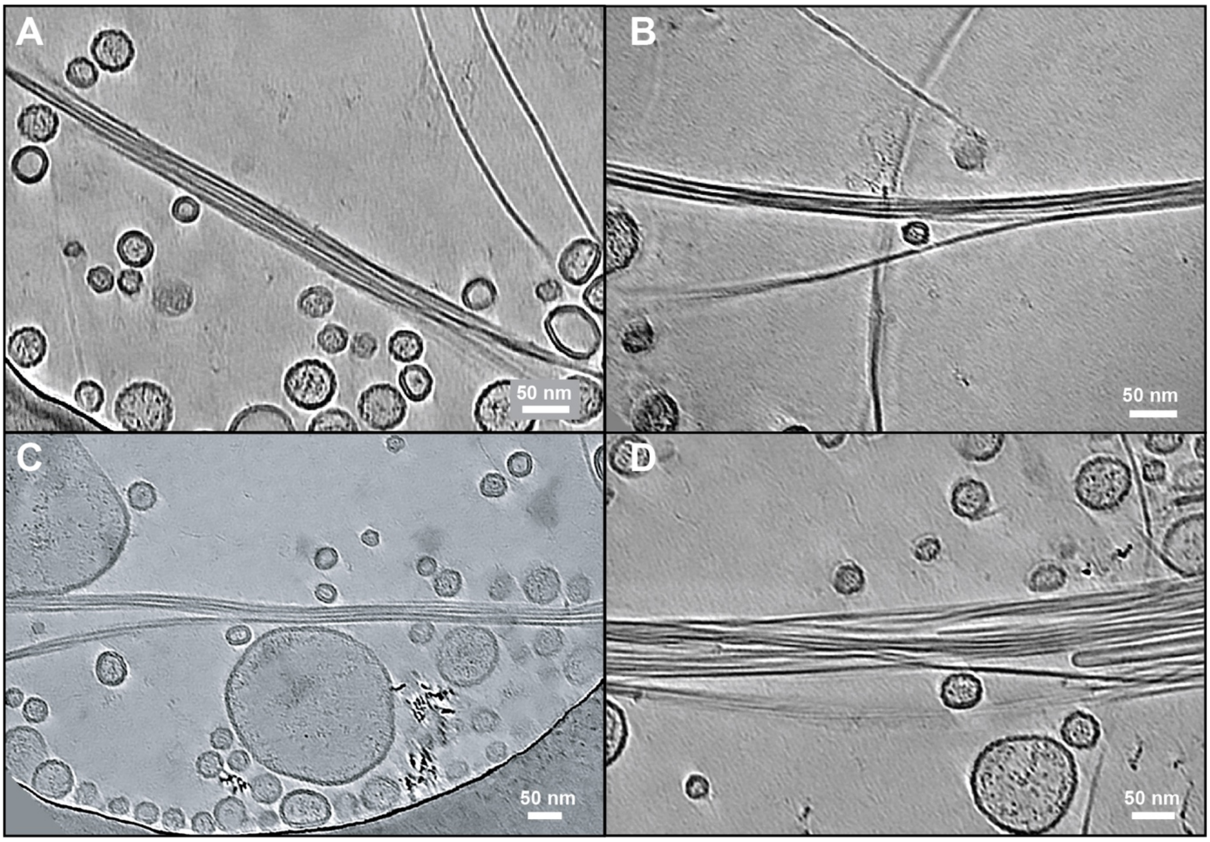

Figure S6: Cryo-ET of MDAΦ bundles.

A-D) Gallery of MDA phage bundles shown as orthogonal slices from denoised tomograms. Vesicle co-purified with MDAΦ can be seen alongside phage bundles.

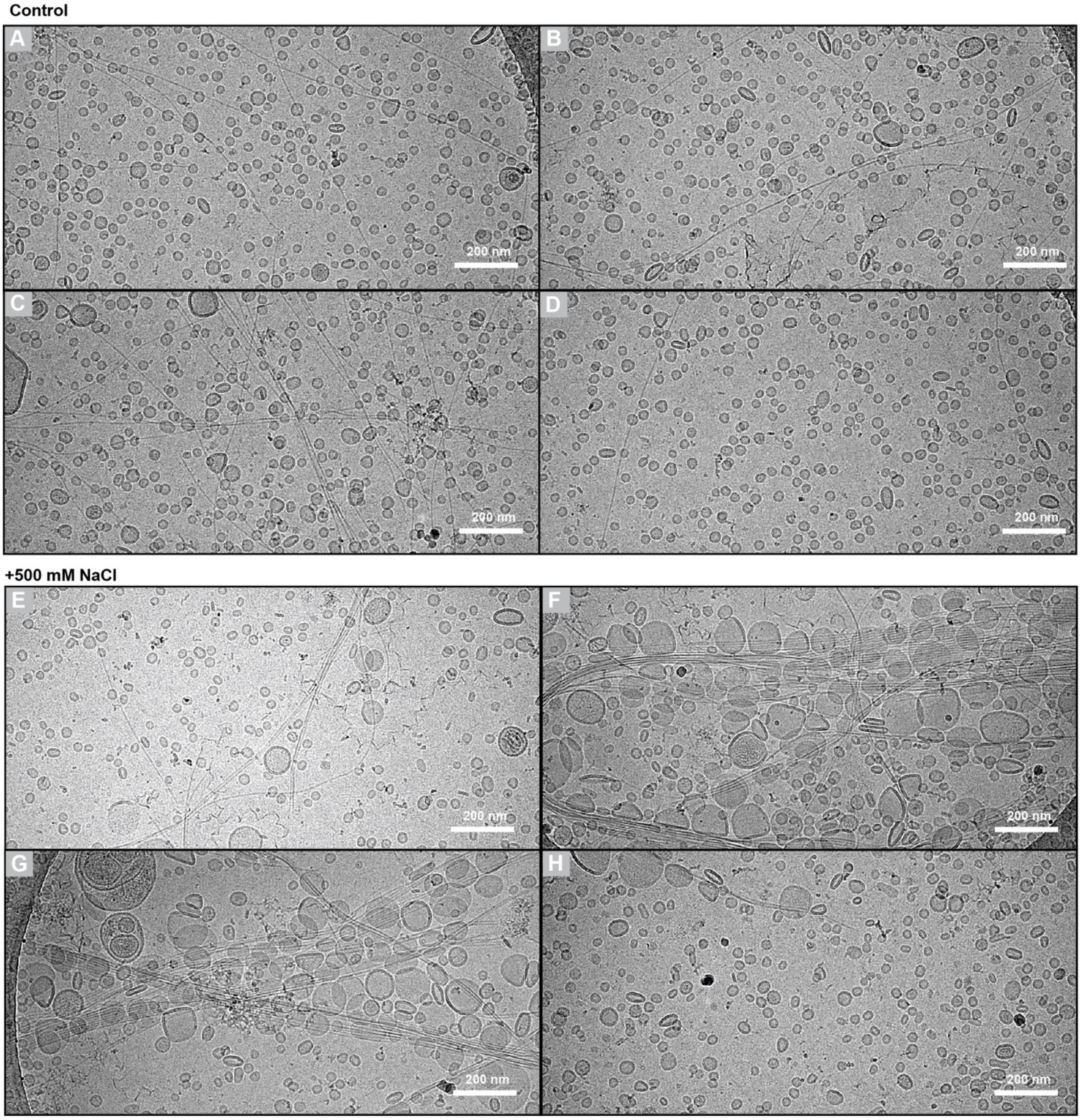

Figure S7: Addition of 500 mM NaCl dramatically increases phage bundling.

Shown are a gallery of micrographs of the MDA phage after the addition of **A-D**) 1:10 PBS (‘control’) or **E-H)** 1:10 5 M NaCl, followed by incubation for 18 hours. While the phage has a propensity to bundle on its own, bundling is dramatically increased when the ionic strength of the medium is increased by salt addition.

Table S1: Cryo-EM data acquisition and processing statistics.

**Data collection and processing**	MDAΦ [EMDB-53129, PDB 9QG9]
Microscope	Krios Titan G2
Magnification	96,000
Voltage (kV)	300
Electron exposure (e^{-}/Å^2)	41.06
Defocus range (μm)	-1 to -2.5
Pixel size (Å)	0.824
Symmetry imposed	C5, helical
Initial particle images (no.)	260,007
Final particle images (no.)	27,134
Map resolution (Å)	3.7
FSC threshold	0.143
Map resolution range (Å)	3.5-6.0
**Model Refinement**	
Initial model used	AlphaFold2
Map sharpening *B* factor (Å^2)	-29.76
Model composition	
Non-hydrogen atoms	1815
Protein residues	255
Ligands	0
B factors (Å^2)	
Protein	174.54
Ligand	n/a
R.m.s. deviations	
Bond lengths (Å)	0.0089
Bond angles (°)	1.77
Validation	
MolProbity score	1.99
Clashscore	4.80
Poor rotamers (%)	2.78
Ramachandran plot	
Favored (%)	93.88
Allowed (%)	6.12
Disallowed (%)	0

Table S2: Genome type (linear versus circular) and number of lysine and arginine residues in the C-termini of the MCPs in experimentally sequenced reference genomes (4).

We propose that the number of C-terminal arginine (R) or lysine (K) residues correlate with genome type, with 3-4 basic residues correlating with a circular genome and 2 basic residues correlating with a linear genome. Experimentally sequenced, reference genomes to which this rule applies are marked in green, while the single reference genome where the rule does not apply is marked in tan colour.

Genome	Protein	Genome	Number of R/K
LC035386	LC035386.BAS49600.1	circ	4
LC210520	LC210520.BAX02602.1	circ	4
NC_001418	NC_001418.NP_040652.1	circ	2
NC_003460	NC_003460.NP_604425.1	circ	4
NC_004306	NC_004306.NP_695190.1	circ	4
NC_004736	NC_004736.NP_835475.1	circ	4
NC_012757	NC_012757.YP_002925191.1	circ	4
AB931172	AB931172.BAP81896.1	circ	3
AY324828	AY324828.AAQ94685.1	linear	2
CP017895	CP017895.ARP06761.1	circ	4
GQ153916	GQ153916.ACY07131.1	circ	4
GQ153919	GQ153919.ACY07161.1	circ	4
LC066596	LC066596.BAS04448.1	circ	3
NC_001331	NC_001331.NP_039603.1	linear	2
NC_001332	NC_001332.NP_039620.1	circ	4
NC_001396	NC_001396.NP_536674.1	circ	3
NC_001954	NC_001954.NP_047355.1	circ	4
NC_002014	NC_002014.NP_040575.1	circ	4
NC_003287	NC_003287.NP_510890.1	circ	4

NC_003327	NC_003327.NP_752644.1	circ	3
NC_005948	NC_005948.YP_031682.1	circ	4
NC_008575	NC_008575.YP_863924.1	circ	3
NC_015297	NC_015297.YP_004327583.1	circ	3
NC_021562	NC_021562.YP_008130278.1	circ	4
NC_021866	NC_021866.YP_008320409.1	circ	3
NC_023586	NC_023586.YP_009008129.1	circ	4
NC_025824	NC_025824.YP_009111302.1	circ	4
AB572858	AB572858.BAJ12070.1	circ	4
AF452449	AF452449.AAL40839.1	circ	4

Movie captions

Movie S1: Cryo-EM structure of the MDAΦ capsid.

Cryo-EM density and ribbon depiction of the atomic model of the MDAΦ capsid are shown.

Movie S2: Electron cryotomography of MDAΦ bundles.

Shown are sequential Z-slices of a tomogram of an MDAΦ bundle.

SI References

1. R. Conners *et al.*, Cryo-electron microscopy of the f1 filamentous phage reveals insights into viral infection and assembly. *Nat Commun* **14**, 2724 (2023).
2. A. K. Tarafder *et al.*, Phage liquid crystalline droplets form occlusive sheaths that encapsulate and protect infectious rod-shaped bacteria. *Proceedings of the National Academy of Sciences* **117**, 4724-4731 (2020).
3. J. Böhning *et al.*, Biophysical basis of filamentous phage tactoid-mediated antibiotic tolerance in *P. aeruginosa*. *Nat Commun* **14**, 8429 (2023).
4. S. Roux *et al.*, Cryptic inoviruses revealed as pervasive in bacteria and archaea across Earth's biomes. *Nature Microbiology* **4**, 1895-1906 (2019).
